# Fractal analysis of nuclear histology integrates tumor and stromal features into a single prognostic factor of the oral cancer microenvironment

**DOI:** 10.1186/s12885-015-1380-0

**Published:** 2015-05-15

**Authors:** Pinaki Bose, Nigel T Brockton, Kelly Guggisberg, Steven C Nakoneshny, Elizabeth Kornaga, Alexander C Klimowicz, Mauro Tambasco, Joseph C Dort

**Affiliations:** 1 Department of Oncology, University of Calgary, Calgary, Canada; 2 Current Address: Canada’s Michael Smith Genome Sciences Centre, British Columbia Cancer Agency, Vancouver, British Columbia Canada; 3 Department of Cancer Epidemiology and Prevention Research, CancerControl Alberta, Alberta Health Services, Calgary, Alberta T2N 2T9 Canada; 4 Department of Anatomic Pathology, Calgary Laboratory Services, Rockyview General Hospital, Calgary, Alberta T2V 1P9 Canada; 5 Department of Surgery, Division of Otolaryngology-Head and Neck Surgery, University of Calgary, Calgary, Alberta T2N 4Z6 Canada; 6 Functional Tissue Imaging Unit, Translational Laboratories, Tom Baker Cancer Centre, Calgary, Alberta T2N 4N2 Canada; 7 Immunology and Inflammation Research, Boehringer Ingelheim Pharmaceuticals, Inc, Ridgefield, Connecticut 06877 USA; 8 Department of Physics, San Diego State University, San Diego, California 92182-1233 USA

## Abstract

**Background:**

The lack of prognostic biomarkers in oral squamous cell carcinoma (OSCC) has hampered treatment decision making and survival in OSCC remains poor. Histopathological features are used for prognostication in OSCC and, although useful for predicting risk, manual assessment of histopathology is subjective and labour intensive. In this study, we propose a method that integrates multiple histopathological features of the tumor microenvironment into a single, digital pathology-based biomarker using nuclear fractal dimension (nFD) analysis.

**Methods:**

One hundred and seven consecutive OSCC patients diagnosed between 1998 and 2006 in Calgary, Canada were included in the study. nFD scores were generated from DAPI-stained images of tissue microarray (TMA) cores. Ki67 protein expression was measured in the tumor using fluorescence immunohistochemistry (IHC) and automated quantitative analysis (AQUA®). Lymphocytic infiltration (LI) was measured in the stroma from haematoxylin-eosin (H&E)-stained TMA slides by a pathologist.

**Results:**

Twenty-five (23.4%) and 82 (76.6%) patients were classified as high and low nFD, respectively. nFD was significantly associated with pathological tumor-stage (pT-stage; *P =* 0.01) and radiation treatment (RT; *P =* 0.01). High nFD of the total tumor microenvironment (stroma plus tumor) was significantly associated with improved disease-specific survival (DSS; *P* = 0.002). No association with DSS was observed when nFD of either the tumor or the stroma was measured separately. pT-stage (*P =* 0.01), pathological node status (pN-status; *P =* 0.02) and RT (*P =* 0.03) were also significantly associated with DSS. In multivariate analysis, nFD remained significantly associated with DSS [HR 0.12 (95% CI 0.02-0.89, *P =* 0.04)] in a model adjusted for pT-stage, pN-status and RT. We also found that high nFD was significantly associated with high tumor proliferation (*P* < 0.0001) and high LI (*P* < 0.0001), factors that we and others have shown to be associated with improved survival in OSCC.

**Conclusions:**

We provide evidence that nFD analysis integrates known prognostic factors from the tumor microenvironment, such as proliferation and immune infiltration, into a single digital pathology-based biomarker. Prospective validation of our results could establish nFD as a valuable tool for clinical decision making in OSCC.

**Electronic supplementary material:**

The online version of this article (doi:10.1186/s12885-015-1380-0) contains supplementary material, which is available to authorized users.

## Background

Almost 30 000 individuals are diagnosed with oral squamous cell carcinoma (OSCC) each year in North America and approximately 6000 of these patients succumb to the disease, annually [1]. OSCC is an aggressive disease and even favourable treatment outcomes are associated with significant morbidity. Five-year survival rates for OSCC have remained between 40 and 50% for the past several decades. Biomarkers that can identify aggressive disease at diagnosis and inform treatment decisions might improve survival outcomes and quality of life for OSCC patients. Although several prognostic markers for OSCC have been described in the literature, treatment is directed predominantly by the tumor-node-metastasis (TNM) staging system.

The tumor microenviroment is a dynamically interacting entity composed of tumor cells and the surrounding stroma. These interactions are not only critical for tumor growth and progression but also for treatment sensitivity/resistance. Therefore, effective prognostic biomarkers should ideally incorporate features of both tumor and stroma, leading to a more comprehensive assessment of tumor biology. Histopathological features have been previously used for prognostication in OSCC. Brandwein-Gensler and colleagues described a histologic risk assessment score based on pattern of invasion (POI), perineural invasion (PNI) and lymphocytic infiltration (LI) [2,3]. These authors propose that achieving negative resection margins do not guarantee local disease-free and overall survival benefits. On the other hand, a combination of histopathological features of the tumor (POI and PNI) and stroma (LI) accurately predicted risk of local recurrence and survival. Although the manual assessment of histological features is a powerful technique for predicting risk, it requires expert subject knowledge of head and neck histopathology and can be very labour intensive. Since few diagnostic laboratories have access to specialized head and neck pathologists, integration of histological feature analysis into a single, digital histopathology-based biomarker may improve the utility and encourage clinical adoption of this type of prognostic testing.

Fractal dimension (FD) is a mathematical measure of the irregularity and complexity of a shape and may be used for the digital assessment and quantification of histological features in the tumor microenvironment [4]. In contrast to our intuitive notion of dimension (i.e. the topological dimension), which is an integer value (0 for a point, 1 for a line, 2 for a plane, etc.), the FD can be a non-integer value that is greater than the topological dimension. The extent to which the FD of an object may be greater than the topological dimension depends on the space filling capacity of the object. FD is a non-integer number that quantifies the degree of space filling of an object. True mathematical fractals exhibit a higher degree of space filling because they exhibit exact or statistical self-similarity in structural patterns when examined to infinitely small scales. As such, actual fractals do not exist in nature, since there is a fundamental natural limitation to the scaling behaviour of natural objects [5]. However, FD analysis has found widespread use in medical image analysis because it lends itself naturally to the pragmatic characterization of irregular non-Euclidean structures found in medical images [6,7]. One such application of FD has been to discriminate the architectural complexity of biological structures associated with neoplastic states. Previous studies have applied FD analysis for the diagnosis, staging and prognosis of several cancer-types including breast [4,8], prostate [9,10], colon [11], lung [12], endometrial [13], gall bladder [14], larynx [15] and OSCC [16]. FD analysis of nuclear histology digitally quantifies the space filling properties of nuclei. Such analysis, when performed on the entire tumor microenvironment (tumor and stroma) can be a source of rich prognostic information.

We have previously used fluorescence immunohistochemistry (IHC) and automated quantitative analysis (AQUA®) to investigate the prognostic value of proteins associated with apoptosis [17,18], proliferation [19] and hypoxia [20,21] in OSCC. We have also reported that the prognostic impact of these biomarkers differs according to their cellular distribution within the tumor microenvironment. Tissue microarrays (TMAs) used to examine protein biomarkers are ideally-suited for digital histological analysis since TMA cores contain both tumor and stromal tissue compartments. When performing AQUA®, images of each whole TMA core are generated and the nuclei are routinely co-stained with 4′,6-diamidino-2-phenylindole (DAPI) to differentiate nuclear/cytoplasmic localization of a biomarker. In this study, we computed the fractal properties of DAPI-stained nuclei in whole TMA cores. We hypothesized that nFD analysis will integrate tumor and stromal characteristics commonly incorporated in OSCC histopathological risk assessment methods into a single, prognostic factor for OSCC. We report that nFD is a robust and powerful independent prognosticator of patient outcome that integrates the proliferative properties of the tumor compartment and the immunologic properties of the stromal compartment into a unified prognostic entity that is amenable to clinical translation.

## Methods

### Patient cohort

This study conforms to the Tri-council Policy Statement for Research with Human Subjects (Canada) and was approved by the University of Calgary Conjoint Health Research Ethics Board. Our retrospective study cohort consisted of 107 histologically confirmed treatment naïve, surgically resected OSCC patients diagnosed between 1998 and 2006 at the Foothills Medical Centre, Calgary, Canada. Eligible patients had no prior history of head and neck cancer. Patients received post-operative radiotherapy based on the presence of metastatic lymph nodes, extra-capsular spread or positive surgical margins. Clinico-pathological characteristics of the patient cohort are described in Table 1.

**Table 1:** **Clinico-pathological characteristics of the patient cohort**

	# Of cases	(%)	nFD low^a^	nFD high^a^	FE*P*- value	DSS (LR*P*- value)
**Number of events**	-	-	33	1	-	-
**Gender**					0.81	0.457
Male	70	65.42	53	17		
Female	37	34.58	29	8		
**Age**	107	NA	61.1^b^	59.8^b^	0.68	nd
**pT-Stage**					**0.01**	**0.01**
pT1/pT2 (low)	61	57.01	41	20		
pT3/pT4 (high)	44	41.12	40	4		
Missing	2	1.87	1	1		
**pN-status**					0.25	**0.02**
pN0	66	61.68	48	18		
pN1/pN2	41	38.32	34	7		
**Smoking history**					0.60	0.27
Never	28	26.168	20	8		
Ever	78	72.897	61	17		
Missing	1	0.935	1	0		
**Alcohol history**					0.43	0.14
Never	11	10.28	10	1		
Ever	57	53.27	43	14		
Missing	39	36.45	29	10		
**Tumor differentiation**					0.23	0.18
Well	15	14.02	10	5		
Moderate	56	52.34	47	9		
Poor	12	11.21	9	3		
Missing	24	22.43	16	8		
**Treatment**					**0.01**	**0.03**
Surgery	34	31.78	20	14		
Surgery + RT	73	68.22	62	11		
**Ki67**						
Low	43	76.19	40	3	**0.001**	**0.002**
High	62	23.81	40	22		
**LI**						
Low	53	50.47	48	5	**0.001**	**0.002**
High	52	49.43	32	20		

^a^Cut-point was determined using X-tile.

^b^Mean age.

nFD: nuclear fractal dimension; pT-stage: pathological T-stage; pN status: pathological node status; RT: radiotherapy; FE: Fisher’s exact; LR: logrank, LI: lymphocytic infiltration. Significant *P* - values are shown in **BOLD**.

### Tissue microarray (TMA) construction

Archived formalin-fixed paraffin-embedded (FFPE) tumor blocks were retrieved for TMA construction. Haematoxylin-eosin (H&E)-stained slides were reviewed by the study pathologist (KG) to select blocks with sufficient tumor content. For each patient included in the study, three 0.6 mm cores were randomly sampled from the tumour-bearing areas of selected FFPE block using a Beecher Manual Tissue Microarrayer (Beecher Instruments Inc. WI, USA). Approximately 100 patients (each with triplicate cores) were included on a TMA block. Slides were assembled using 4 μm thick sections from the TMA block.

### Fractal dimension analysis

TMAs were immunofluorescently stained as previously described [18]. High resolution images of nuclei, defined by positive DAPI-stained regions, for each TMA core were collected for subsequent analysis as part the automated quantitative analysis (AQUA®) process. Images were acquired at 20X magnification corresponding to a resolution of 0.468 μm/pixel and saved in tagged image file format (.tiff) for fractal analysis. Cores were excluded from analysis if they were out of focus, tissue was folded, or there was insufficient tumor present (less than 100 tumor cells).

We applied an automated fractal analysis technique that we developed in previous work [5,10] to quantify the degree of space filling of nuclei. In summary, this technique involves the following steps:

1. Application of a series of intensity thresholds to convert the acquired grey-scale DAPI images (from AQUA®) into a series of binary images to derive the outlines of nuclei.
2. Application of the box counting method (with appropriate spatial scale range for our structures of interest – nominally ~4 to 60 μm) [5,10] to compute the fractal dimension of each outline image obtained from step 3.
3. Identification of the global maximum from a plot of fractal dimension versus intensity threshold. This maximum corresponds to the fractal dimension of the pathological structures [10].

Our automated fractal analysis method was applied to a total of 321 TMA cores (3 cores for each of the 107 patient samples), and for each patient the mean nFD from the three TMA cores was used in statistical analyses.

### Ki67 fluorescence IHC

Ki67 staining has been described previously [19]. Briefly, TMAs were stained for DAPI (Invitrogen), Ki67 (mouse monoclonal, clone MIB1, DAKO) and Pan-cytokeratin (PCK; guinea pig polyclonal, ACRIS). We used the Aperio Scanscope® FL slide scanner for automated fluorescent image acquisition and HistoRx AQUAnalysis® software version 2.3.4.1 for automated image analysis.

### Lymphocytic infiltration

H&E stained OSCC TMA cores were evaluated for the presence of LI at the invasive boundary of the tumor at 200X microscope magnification by the study pathologist (KG). All mononuclear cells including lymphocytes and plasma cells were scored (granulocytes and other polymorphonuclear leukocytes were excluded). Necrotic areas near the invasive tumor boundaries were excluded from LI assessment. LI was classified as a four-tiered variable: zero infiltration, weak infiltration, intermediate infiltration and strong infiltration. For each patient, the maximum value of the LI among three TMA cores was used for statistical analyses.

### Statistical analysis

X-Tile version 3.6.1 software was used to determine optimal cut-points to dichotomize continuous nFD scores [22]. In Table 1, Fisher’s exact test was used to compare clinical covariates between the two patient groups defined by low or high nFD. Kaplan-Meier curves and Cox proportional hazards models were used to assess association with 5-year disease-specific survival (DSS). Clinical covariates that are usually associated with prognosis in OSCC such as pathological T-stage (pT-stage) and pathological node status (pN-status) were subjected to Cox univariate analysis. Clinical covariates that were significantly associated with DSS in univariate analysis were included in a multivariate model with nFD. In all analyses, a *P*-value of < 0.05 was considered statistically significant. All statistical analyses were performed using Stata 13 data analysis and statistical software (StataCor*P* LP, College Station, Tx, USA).

## Results

### Cohort characteristics

Our study was conducted and reported according to Reporting recommendations for tumor marker prognostic studies (REMARK) criteria for reporting tumor biomarker prognostic studies [23]. The median age at diagnosis for the study cohort was 62.35 years (range: 25.73 – 95.12 years). Median survival was 77.85 months; survivor follow-up duration ranged between 1.3 and 156.9 months [standard deviation = 33.3]. All patients were treated with primary surgery and 73 (68.2%) received post-operative radiotherapy. Univariate analyses of the association of clinico-pathological variables with DSS are presented in Table 1. pT-stage (*P* = 0.01), pN-status (*P* = 0.02) and treatment (whether patients received post-operative radiotherapy; *P* = 0.03) were significantly associated with DSS. Patients with high nFD did not differ significantly from patients with low nFD in terms of age of diagnosis, gender, pN-status, smoking history, alcohol history and tumor differentiation status (Fisher’s exact test).

### Fractal analysis of nuclei and survival analyses

**Figure 1:**
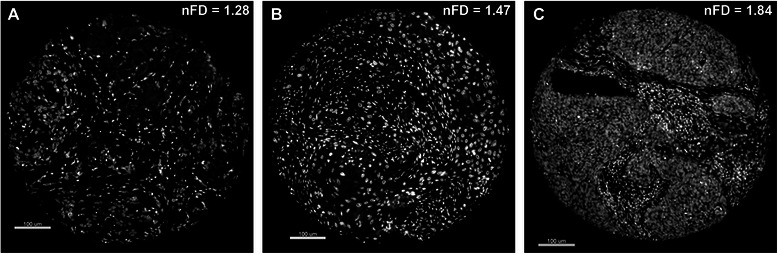
Representative DAPI-stained images of individual tissue microarray (TMA) cores used as substrates for nuclear fractal dimension (nFD) analysis. Image of an entire TMA core with **(A)** low nFD, **(B)** intermediate nFD and **(C)** high nFD.

**Figure 2:**
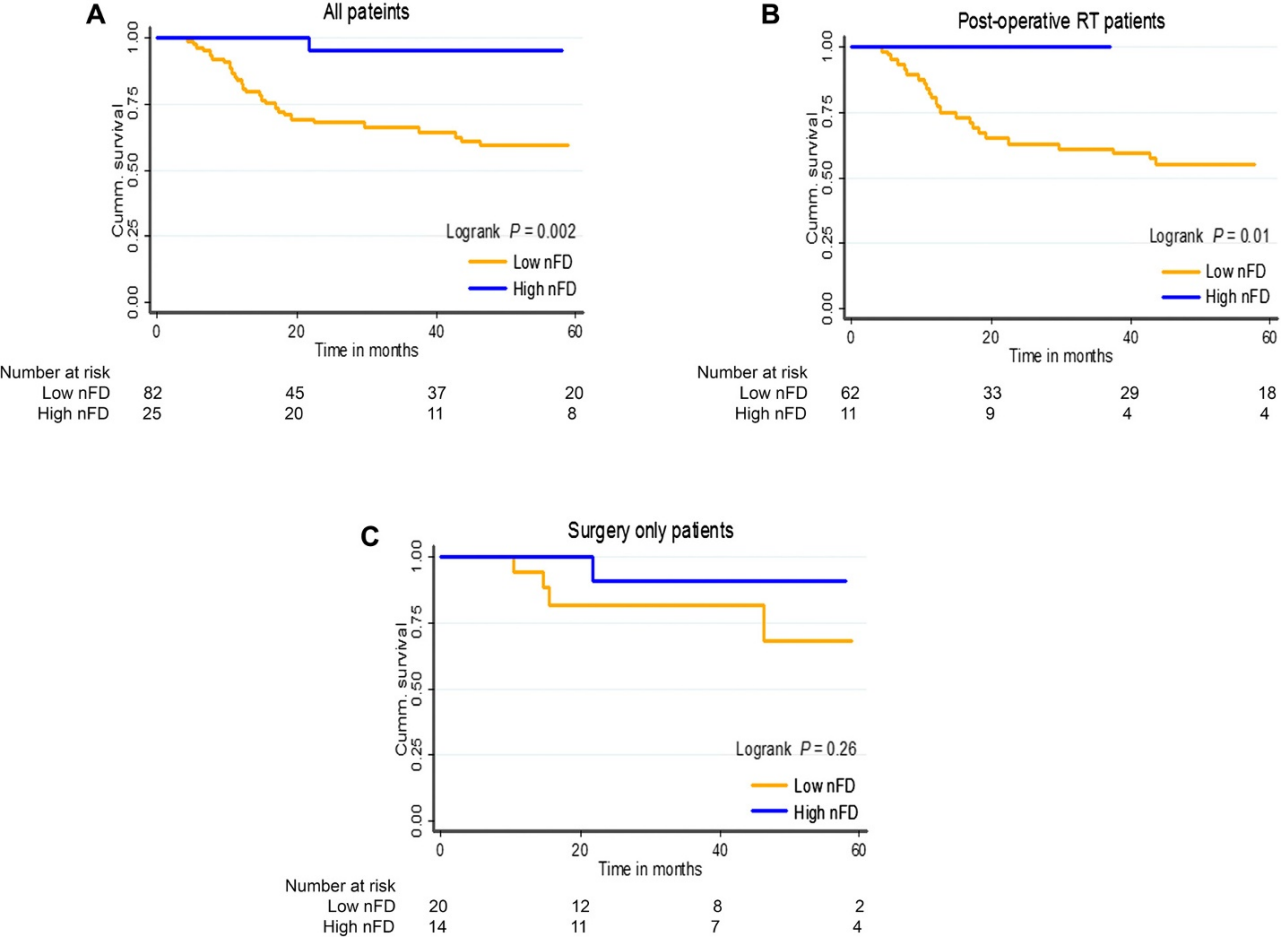
Five-year disease-specific survival (DSS) in OSCC patients stratified by nuclear fractal dimension (nFD). Kaplan-Meier curves for DSS by nFD in **(A)** all patients, **(B)** patients who received radiotherapy after surgery and **(C)** patients who did not receive post-operative radiotherapy.

nFD scores ranged between 1.19 and 1.84, with a median of 1.52, lower quartile 1.42, and upper quartile 1.64. Figure 1 shows representative monochromatic DAPI-stained images of TMA cores with low (1.28), intermediate (1.47) and high (1.84) nFD. All TMA cores examined contained nuclei from both the tumor and the stromal tissue compartments. Twenty-five (23.4%) patients were classified as high nFD and 82 (76.6%) were classified as low nFD. Among the clinical covariates assessed, high pT-stage was significantly associated with low nFD scores (Fisher’s Exact *P* = 0.01; Table 1). Also, most patients who received post-operative radiotherapy had low nFD scores (Fisher’s Exact *P* = 0.01; Table 1). In our entire cohort of 107 OSCC patients, high nFD was associated with significantly better DSS compared to low nFD (Figure 2A); the HR estimate was 0.09 (95% CI, 0.01 to 0.64), reflecting a 91% reduction in DSS non-achievement in patients with high nFD (*P* = 0.02; Table 2). A significant association between nFD and DSS was also observed when the analysis was restricted to patients who received post-operative radiotherapy (73 patients; logrank *P* = 0.01; Figure 2B); no association between nFD and DSS was observed in patients who were treated with surgery alone (logrank *P* = 0.26; Figure 2C). Furthermore, nFD remained an independent prognostic factor in our OSCC cohort [HR 0.11 (95% CI, 0.02 to 0.83), *P* = 0.03] after adjusting for other known prognostic factors including pT-stage [HR 2.26 (95% CI 1.10 to 4.50, *P* = 0.02)] and pN-status [HR 2.14 (95% CI 1.10 to 4.30, *P* = 0.03)] (Table 2). The nFD of the tumor compartment or stromal compartment alone were not significantly associated with survival (data not shown).

**Table 2:** **Univariate and multivariate analysis of 5-Year Disease-Specific Survival (DSS)**

	Univariate			Multivariate		
	HR	95% CI	*P*- value	HR	95% CI	*P*- value
nFD (low vs. high)	0.09	0.01 - 0.64	**0.02**	0.11	0.02 – 0.83	**0.03**
pT-stage (T1/T2 vs. T3/T4)	2.83	1.42 – 5.67	**0.003**	2.26	1.10 – 4.50	**0.02**
pN-status (N0 vs. N1/N2)	2.19	1.12 - 4.31	**0.02**	2.14	1.10 – 4.30	**0.03**

HR: hazard ratio; CI: confidence interval. HRs estimated from stratification of Cox proportional hazard models. Significant *P* - values are shown in **BOLD**.

### Association between nFD and tumor proliferation

**Figure 3:**
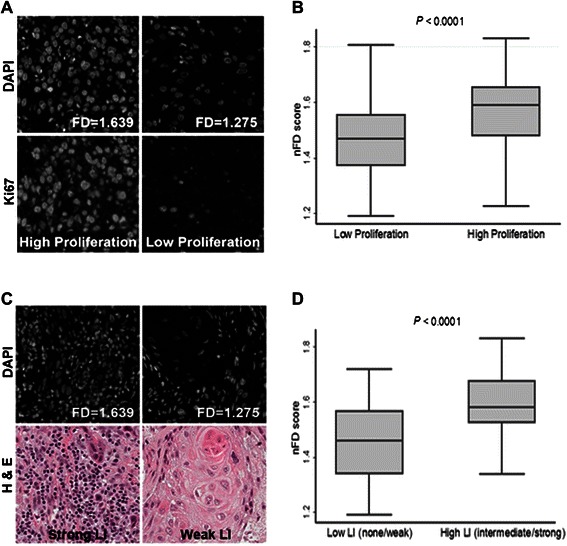
Association between nuclear fractal dimension (nFD) and features of the tumor microenvironment. **(A)** Representative DAPI-stained images of TMA cores with high and low nFD (upper panels) and images of the same cores stained for Ki67 (lower panels). **(B)** Box and whisker plot showing the association between nFD and tumor proliferation. **(C)** Representative DAPI-stained images of TMA cores with high and low nFD (upper panels) and H & E-stained images of cores from the same patient that were used for assessing lymphocytic infiltration (LI; lower panels). **(D)** Box and whisker plot showing the association between nFD and LI.

We have previously reported that increased tumor cell proliferation in OSCC is associated with significantly better survival that may be attributed to an improved response to post-operative radiotherapy [19]. Tumor proliferation was assessed by Ki67 staining of nuclei in the PCK-stained tumor compartment. Figure 3A shows representative images of DAPI-stained nuclei from TMA cores within the PCK-stained tumor compartment and corresponding H&E-stained slides from the same patient. The box and whisker plots (Figure 3B) illustrate that the mean nFD was significantly higher in the high proliferative index group (*P* < 0.0001).

### Association between nFD and LI

In order to better understand how nFD is correlated with characteristics of the tumor microenvironment, we studied the association between nFD and stromal LI. Figure 3C shows representative fluorescent images of DAPI-stained nuclei from TMA cores in the PCK-negative stromal compartment with corresponding nFD scores (upper panel) and H&E-stained TMA cores from the same patients showing LI (lower panel). Considerable heterogeneity in terms of LI scores was observed among TMA cores from the same patient, ranging from weak infiltration in one core to strong infiltration in another. The core showing the maximum infiltration was used as the representative core for each patient. As evident from the box and whisker plots (Figure 3D), nFD values were positively correlated with increased LI in the stroma (*P* < 0.0001). In agreement with previous reports [24-26], high LI was associated with significantly improved survival in our OSCC cohort (*P* = 0.001; Additional file 1: Figure S1).

## Discussion

We report a digital histopathologal image-based prognostic biomarker (nFD) derived from fractal analysis of DAPI-stained nuclei. This single measure integrates features of both the stromal and tumor compartments in the tumor microenvironment. nFD can effectively discriminate between OSCC patients with good and worse prognosis and was an independent prognostic indicator in our OSCC cohort when the model was adjusted for established prognostic clinical covariates. A strong positive correlation was observed between nFD and a pathologist-scored assessment of LI in the stroma. nFD was also positively correlated with proliferation (scored using fluorescence IHC and AQUA®), an important tumor-associated prognostic marker. The significance of both these features, independently, suggests that nFD scores are an effective method for the automated, image-based, integration of both stromal and tumor features with acknowledged prognostic value.

OSCC is a serious public health problem worldwide and the lack of effective prognostic biomarkers adversely affects patient management and survival outcomes. This has led researchers to look beyond the traditional TNM staging system and investigate biological correlates to histopathological features of the tumor microenvironment. Staining with DAPI is a routine component of IHC that helps identify nuclei. We hypothesized that FD analysis of DAPI-stained images (computer-acquired when performing AQUAnalysis®), could provide a digital histology-based prognostic factor for OSCC that might be more objective and less labour-intensive than traditional histopathological analysis. FD analysis has been previously used in OSCC. Several researchers have demonstrated that FD can discriminate between normal versus malignant oral tissue [27,28]. Goutzanis and colleagues have used FD to assess vascularization and also nFD as a prognostic factor [16,29]. However, contrary to our results, these authors report that high nFD is associated with poor prognosis [16]. It is worth noting that almost all previously reported studies have used 3,3′-dichlorobenzidine (DAB) IHC-based images for FD analysis. Also, these studies did not take into account the tumor microenvironment that might provide valuable biologic information relevant to prognosis. DAPI-based nuclear staining is more robust than DAB IHC-based techniques since it is not affected by antibody specificity issues. Also DAPI staining is relatively easy to perform that protein-based IHC since protein is more sensitive than DNA to pre-analytical variables, particularly when the motive is to preserve overall DNA structure rather than specific base pairs. Also, DAPI staining allows for multiplexing of diverse stains, allowing for staining of additional proteins that can, e.g. discriminate the tumor (PCK) and stromal (vimentin) compartments [21]. Interestingly, we found that nFD scores from the stromal or tumor compartment alone did not show a significant association with survival. However, a robust association with survival was observed when nFD from the whole tissue core (tumor plus stroma) was considered.

In order to understand how well digital nFD-based histopathological analysis correlates with expert pathological assessment of stromal morphological parameters associated with survival, we compared LI, scored by a pathologist, with nFD scores. We also studied the relationship between nFD and tumor proliferation in order to evaluate if nFD correlated with tumor-associated prognostic features. LI has been previously reported to be associated with prognosis in OSCC [24-26] and was significantly associated with both DSS and OS in our OSCC cohort as well (Additional file 1: Figure S1). We observed that high nFD was associated with increased LI in the stroma and cell proliferation in the tumor. We believe that high nFD tumor and stroma might reflect increased proliferation in the tumor and the presence of infiltrating immune cells in the stroma. Both have been shown to be associated with improved prognosis attributed to increased susceptibility to radiotherapy. We found that patients with high nFD scores have significantly better survival when treated with post-operative radiotherapy compared to patients with low nFD (Figure 3b). Although further investigation is required, nFD analysis may represent a summary measure incorporating tumor proliferation and the immune involvement that predicts response to radiotherapy [30]. Therefore, high nFD might represent a state where high proliferation renders tumor cells sensitive to radiotherapy and dying cells are cleared by the infiltrating immune cells, creating a “perfect storm” for the tumor.

## Conclusions

The histopathological scoring system proposed by Brandwein-Gensler et al. is a powerful tool but its broad implementation may be limited by its labour intensiveness and the requirement for extensive training. Additionally, consistency of scoring is a substantial risk given the high degree of inter-observer variability in other oral histopathology-based systems, among pathologists [31,32]. Digital pathology-based nFD scoring incorporates multiple biomarkers and therefore might provide a more reliable and objective indicator of prognosis compared to single biomarker-based assays. We believe that a comprehensive approach to the analysis of tumor microenvironment, such as the one presented here, will improve prognostication and outcomes in OSCC.

### Consent

Written informed consent was obtained from the patient for the publication of this report and any accompanying images.

## Additional file

Additional file 1: Figure S1. Five-year disease-specific survival (DSS) in OSCC patients stratified by lymphocytic infiltration (LI). Kaplan-Meier curves for DSS in patients stratified by high and low (LI).

## Abbreviations

AQUA: Automated quantitative analysis
CI: Confidence interval
DAB: 3,3′-dichlorobenzidine
DAPI: 4′,6-diamidino-2-phenylindole
DSS: Disease-specific survival
FFPE: Formalin-fixed, paraffin–embedded
H&E: Haematoxylin-eosin
HR: Hazard ratio
IHC: Immunohistochemistry
LI: Lymphocytic infiltration
nFD: Nuclear fractal dimension
OSCC: Oral squamous cell carcinoma
PCK: Pan-cytokeratin
PNI: Perineural invasion
POI: Pattern of invasion
pN-status: Pathological node status
pT-stage: Pathological T-stage
REMARK: Reporting recommendations for tumor marker prognostic studies
RT: Radiation treatment
TMA: Tissue microarray
TNM: Tumor-node-metastasis
