# Supplementary figures and images for: Fractal analysis of nuclear histology integrates tumor and stromal features into a single prognostic factor of the oral cancer microenvironment
Source: BMC Cancer. 2015 May 15;15:409. doi: 10.1186/s12885-015-1380-0 (PMC4435912; doi:10.1186/s12885-015-1380-0)

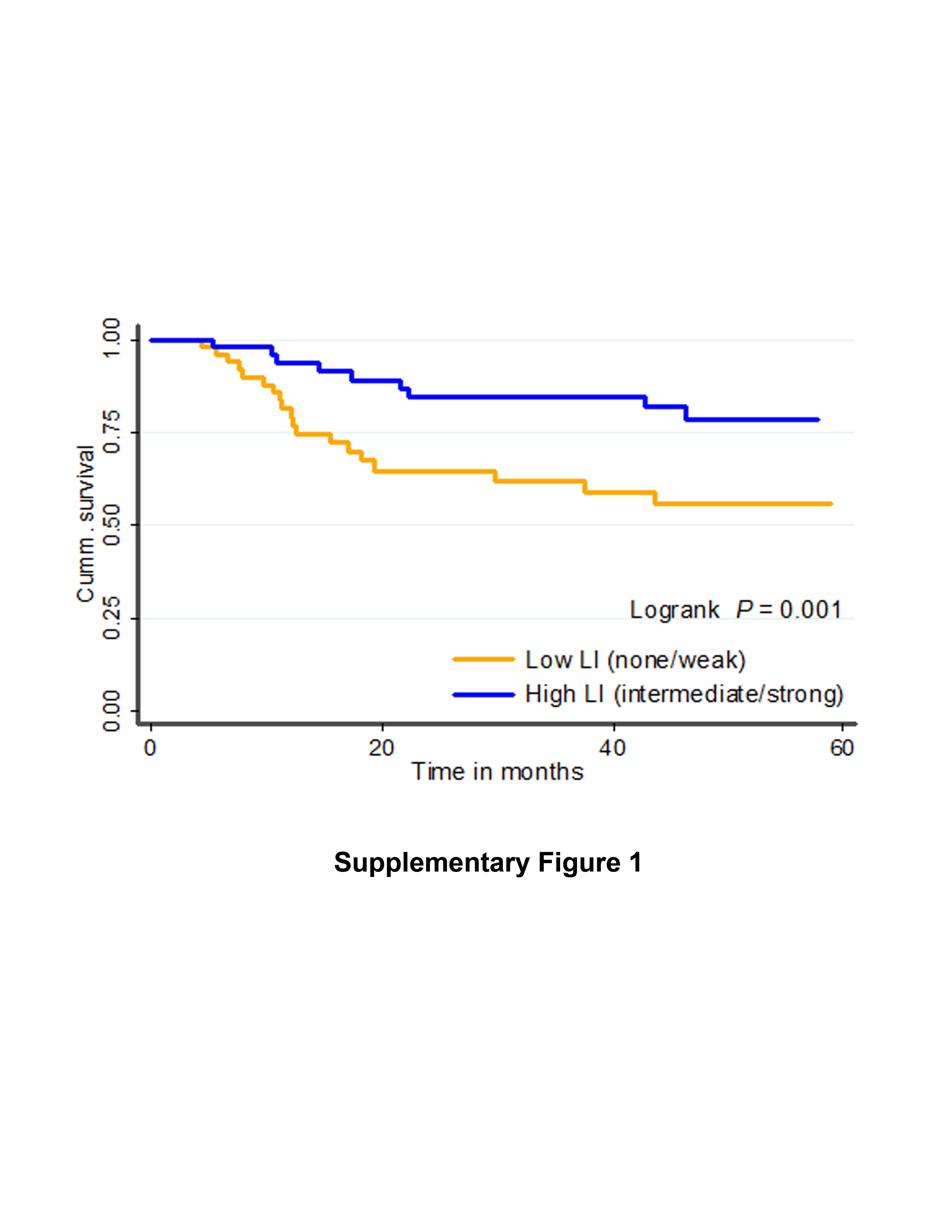

Supplement: Additional file 1: Figure S1. — Five-year disease-specific survival (DSS) in OSCC patients stratified by lymphocytic infiltration (LI). Kaplan-Meier curves for DSS in patients stratified by high and low (LI). [file 12885_2015_1380_MOESM1_ESM.jpeg]
